# Suppress Me if You Can: Neurofeedback of the Readiness Potential

**DOI:** 10.1523/ENEURO.0425-20.2020

**Published:** 2021-03-08

**Authors:** Matthias Schultze-Kraft, Vincent Jonany, Thomas Samuel Binns, Joram Soch, Benjamin Blankertz, John-Dylan Haynes

**Affiliations:** 1 Bernstein Center for Computational Neuroscience Berlin, Charité - Universitätsmedizin Berlin, corporate member of Freie Universität Berlin, Humboldt-Universität zu Berlin, and Berlin Institute of Health, Berlin 10117, Germany; 2 Berlin Center for Advanced Neuroimaging, Charité - Universitätsmedizin Berlin, corporate member of Freie Universität Berlin, Humboldt-Universität zu Berlin, and Berlin Institute of Health, Berlin 10117, Germany; 3 SFB 940 Volition and Cognitive Control, Technische Universität Dresden, Dresden 01069, Germany; 4 Neurotechnology Group, Technische Universität Berlin, Berlin 10587, Germany; 5 School of Medicine, Medical Sciences and Nutrition, University of Aberdeen, Aberdeen AB24 3FX, United Kingdom; 6 German Center for Neurodegenerative Diseases, Göttingen 37075, Germany; 7 Clinic of Neurology, Charité - Universitätsmedizin Berlin, corporate member of Freie Universität Berlin, Humboldt-Universität zu Berlin, and Berlin Institute of Health, Berlin 10117, Germany; 8 Department of Psychology, Humboldt Universität zu Berlin, Berlin 12489, Germany; 9 Cluster of Excellence Science of Intelligence, Technische Universität Berlin und Humboldt Universität zu Berlin, Berlin 10117, Germany

**Keywords:** conscious control, EEG, neurofeedback, readiness potential, voluntary movement

## Abstract

Voluntary movements are usually preceded by a slow, negative-going brain signal over motor areas, the so-called readiness potential (RP). To date, the exact nature and causal role of the RP in movement preparation have remained heavily debated. Although the RP is influenced by several motorical and cognitive factors, it has remained unclear whether people can learn to exert mental control over their RP, for example, by deliberately suppressing it. If people were able to initiate spontaneous movements without eliciting an RP, this would challenge the idea that the RP is a necessary stage of the causal chain leading up to a voluntary movement. We tested the ability of participants to control the magnitude of their RP in a neurofeedback experiment. Participants performed self-initiated movements, and after every movement, they were provided with immediate feedback about the magnitude of their RP. They were asked to find a strategy to perform voluntary movements such that the RPs were as small as possible. We found no evidence that participants were able to to willfully modulate or suppress their RPs while still eliciting voluntary movements. This suggests that the RP might be an involuntary component of voluntary action over which people cannot exert conscious control.

## Significance Statement

The readiness potential (RP), a brain signal that precedes spontaneous, voluntary movements, has been a matter of controversial research for several decades. There has been a long debate on the nature of this signal and the degree to which it undermines the control a person has over their behavior. Thus, assessing the degree to which people are able to exert control over this brain signal is of vital importance. We addressed this question in a neurofeedback experiment. Our results show that people are unable to willfully suppress their RPs, even when explicitly trying to do so. This suggests that the RP is an involuntary and irrevocable component of voluntary action over which people have no control.

## Introduction

The readiness potential (RP) is a slow scalp negativity observed over motor areas in the electroencephalogram (EEG) and can start >1 s before spontaneous, voluntary movements (Kornhuber and Deecke, 1965; Shibasaki and Hallett, 2006). One traditional account of the RP is that it is a causal precursor to voluntary action and that it reflects an unconscious decision to act (Libet et al., 1983; Libet, 1985). While recent studies indeed suggest that the RP is involved in the formation of conscious intention (Parés-Pujolràs et al., 2019; Schultze-Kraft et al., 2020) and that it is a signal specific to voluntary action (Travers et al., 2020), other studies have raised questions about its role in movement preparation (Schurger et al., 2012; Schmidt et al., 2016; Schurger, 2018) and its role in human volition has remained unclear (Frith and Haggard, 2018).

The precise causal role of the RP in movement preparation notwithstanding, it is frequently assumed that it is a necessary part of the causal chain that allows for voluntary action (although this is debated, see Radder and Meynen, 2012). A related and more specific possibility could be that the RP is an “involuntary component of voluntary action.” That is, that the RP occurs automatically and irreversibly (i.e., involuntarily) once a person has voluntarily decided to move. In contrast, an alternative possibility is that people can exert conscious control over their RP, for example, by learning to suppress or abolish it completely, while still being able to elicit spontaneous movements. This possibility has not yet been tested directly. One way to test this would be to provide people with immediate and graded neural feedback about the size of the RP they just produced and ask them to reduce it. Such an approach could potentially enable people in a trial-and-error fashion to learn how to modulate and suppress their RPs, as with examples of neurofeedback for other cognitive processes (Papo, 2019).

We distinguish two principles which could enable people to achieve control over their RP. First, the RP has been shown to be modulated by various attributes of voluntary movement, such as its inertial load and force deployment (Becker and Kristeva, 1980; Kristeva et al., 1990; Slobounov et al., 2004), its complexity (Benecke et al., 1985; Simonetta et al., 1991; Kitamura et al., 1993), its purposiveness and selection mode (Praamstra et al., 1995; Masaki et al., 1998), and by explicit demands on timing (Bortoletto and Cunnington, 2010; Baker et al., 2012; Verleger et al., 2016). Further, compared with RPs observed in classical Libet-style studies (Libet et al., 1983), RPs are considerably smaller when spontaneous movements are executed unconsciously (Keller and Heckhausen, 1990), and almost absent when movements are initiated by deliberate, value-based decisions (Maoz et al., 2019). In all these studies, the modulation of the RP resulted from an experimental manipulation, that is by instructing participants to change specific characteristics of voluntary movements. However, it seems plausible that, when provided with trial-by-trial feedback of their RP, people would be able to identify how changing specific movement features allows them to modulate their RPs.

Second, studies have investigated the self-regulation of slow cortical potentials (SCPs), which are polarizations of EEG that can last up to several seconds (Birbaumer, 1999), and of which RPs are considered a specific type. Using a training based on visual feedback of SCP shifts and operant learning principles (Elbert et al., 1980; Rockstroh et al., 1984), people can learn to self-regulate their SCPs, which has been used in communication systems for paralyzed patients (Kübler et al., 1999, 2001; Neumann et al., 2004). The mechanisms that allow such self-regulation are not well understood but are assumed to be based on a redistribution of attentional resources (Birbaumer, 1999). This learning of self-regulation could in principle be employed by participants when provided with a trial-by-trial feedback of RP magnitude.

Here, we tested the possibility of a voluntary suppression of RPs in a neurofeedback experiment. Our core research question was whether people could suppress RPs by purely mental efforts, and not by changing physical movement characteristics that are known to modulate RPs. Participants performed self-paced pedal presses in single trials. After each pedal press, we used a machine learning approach to derive a score that reflected the size of the RP that had just been produced and that was shown to participants as feedback. Participants were challenged to find a mental strategy to perform movements such that the scores (and therefore their RPs) were as small as possible.

## Materials and Methods

### Participants

Based on the average sample size of previous studies (Schurger et al., 2012; Parés-Pujolràs et al., 2019; Schultze-Kraft et al., 2020), we aimed for a minimum sample size of 15 participants. Considering that some would have to be excluded, we tested a total of 22 participants. Following our exclusion criteria (see section “Data selection”), 19 participants were included in the final sample (11 female, mean age 26.9, SD 5.7 years). The experiment was approved by the local ethics board and was conducted in accordance with the Declaration of Helsinki. All participants gave their informed oral and written consent, and were paid €10 per hour.

### Experimental setup

Participants were seated in a chair facing a computer screen at a distance of ∼1 m. They were asked to place their hands in their lap and to position their right foot to the right of a 10 × 20 cm floor-mounted switch pedal (Marquardt Mechatronik GmbH). Throughout the experiment, EEG was recorded at 1 kHz with a 64-electrode Ag/AgCl cap (EasyCap, Brain Products GmbH) mounted according to the 10–20 system, referenced to FCz and re-referenced offline to a common average. EEG was recorded from the following 51 electrodes: AF7, AF3, Fpz, AF4, AF8, FT7, F5, F3, F1, Fz, F2, F4, F6, FT8, FC5, FC3, FC1, FC2, FC4, FC6, C5, C3, C1, Cz, C2, C4, C6, CP5, CP3, CP1, CPz, CP2, CP4, CP6, TP7, P5, P3, P1, Pz, P2, P4, P6, TP8, PO3, PO1, POz, PO2, PO2, O1, Oz, O2. In order to obtain the earliest measure of movement onset, 3D acceleration of the right leg was recorded with an accelerometer (Brain Products GmbH) that was attached with an elastic band to the right calf. The amplified signal (analog filters: 0.1, 250 Hz) was converted to digital (BrainAmp MR Plus and BrainAmp ExG, Brain Products GmbH), saved for offline analysis, and simultaneously processed online by the Berlin Brain-Computer Interface toolbox (BBCI; https://github.com/bbci/bbci_public). The Pythonic Feedback Framework (Venthur et al., 2010) was used to generate visual feedback.

### Experimental design

**Figure 1.**
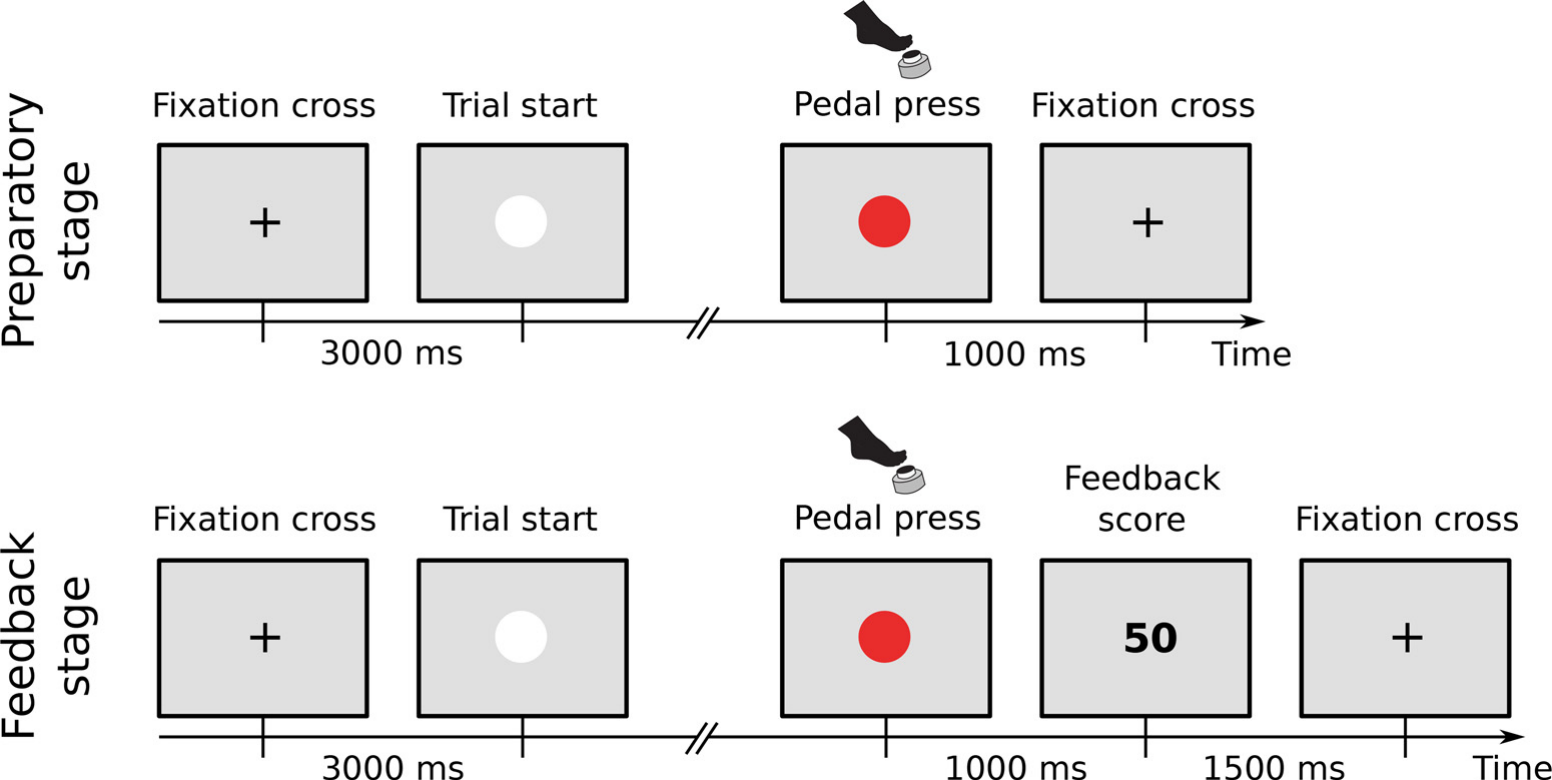
Experiment paradigm. In both the preparatory and the feedback stage, trial start was signaled by a white circle appearing on the screen. When a pedal press was executed, the circle turned red for 1 s. In the preparatory stage, the trial ended and a fixation cross was shown for an intertrial period of 3 s. In the feedback stage, before the trial ended a number was shown on the screen for 1.5 s, after which the fixation cross was shown.

The experiment consisted of two stages (Fig. 1), a preparatory stage, and a feedback stage. The preparatory stage was performed to obtain data for training a classifier in preparation for the subsequent feedback stage. During the preparatory stage participants performed a simple self-paced movement task. The start of a trial was signaled by a white circle appearing on the screen. Participants were instructed to wait for roughly 2 s, after which they could press the pedal at any time. In accordance with standard definitions of the RP they were asked to avoid preplanning the movement, avoid any obvious rhythm, and to press when they felt the spontaneous urge to move (Kornhuber and Deecke, 1965; Libet et al., 1983). When the pedal was pressed the white circle turned red for 1 s, after which it disappeared and was replaced by a fixation cross. This constituted the end of a trial. The fixation cross remained onscreen for a 3-s intertrial period. Each participant performed a total of 100 trials in the preparatory stage, with the possibility of taking a break after each 25 trials.

During the second part of the experiment, the feedback stage, participants again performed self-paced pedal presses in single trials, as during the preparatory stage. However, after the participants had moved, an integer number was displayed on the screen for 1.5 s. Participants were informed that “this number reflects a brain signal recorded when you decided to press the pedal. Larger numbers mean large signals, small numbers mean small signals.” They were given the additional task to develop strategies to achieve preferably low numbers. Participants were (1) instructed to move spontaneously and to not execute abnormal (e.g., very slow, interrupted) movements, but were otherwise free to find a strategy to achieve the goal, (2) informed that, based on noisy measurements, scores might greatly vary from trial to trial and that they might thus need many trials to realize if a strategy works or not, and (3) instructed to keep using a strategy to further lower the scores if they happen to find one that works. Participants performed 300 trials during the feedback stage, with the possibility of taking a break after each 25 trials.

### Training of classifiers from preparatory stage data

Before the feedback stage, we performed three consecutive analyses on the data recorded during the preparatory stage: (1) we trained an accelerometer classifier that detected physical movement onset times in real time from accelerometer data, (2) we selected the most informative EEG channels, and (3) we trained a real-time EEG classifier. Both the accelerometer and EEG classifiers were then used during the feedback stage to assess the movement and the RP produced in each trial and to derive a score in real time that was shown to participants as feedback at the end of the trial.

#### Detection of movement onsets from accelerometer

The accelerometer device attached to the right calf recorded acceleration in the direction of three orthogonal space axes. We determined the time of movement onset in each trial with a variance-based approach. We trained a linear classifier on log-variance features extracted from two time windows: (1) a time window from −200 to 0 ms, time-locked to pedal press (“movement” class), and (2) a time window from 300 to 500 ms, time-locked to trial start (“idle” class). The former time window was expected to contain the acceleration of the foot during the movement, and thus have a large variance, while in the latter the acceleration was expected to be at baseline during the instructed self-paced waiting time (WT) of 2 s. In order to determine the movement onsets of each trial, a classifier was trained on the movement and idle time windows of 99 trials, and then applied with a sliding window on the remaining trial. The analysis worked backward from the physical completion of the pedal press, looking for the last time window preceding the pedal press where there was no evidence for movement. For this, a first window was time-locked to the pedal press and then it was sample-wise shifted back in time until the classifier output indicated being in the idle class. The time of this last idle window before movement was registered as the time of movement onset. This procedure was applied to each of the 100 trials per participant in a leave-one-out scheme. Trials with movement onsets times 3 SDs below or above the individual mean were excluded from further analysis. Finally, a classifier (hereafter referred to as “accelerometer classifier”) was trained on the accelerometer data from all remaining trials and subsequently used during the feedback stage for real-time detection of movement onset (see section “Real-time feedback”).

#### EEG channel selection

We preselected a subset of channels that would be used for the assessment of RP magnitude during the feedback stage. This selection was done using the independent data recorded during the preparatory stage. By selecting channels near the vertex, we focused on channels where the RP is assumed to predominate, and further aimed to minimize the impact of movement or eye artifacts that predominantly occur at peripheral electrodes. For each of the selected trials of the preparatory stage, we defined two EEG segments: (1) a 1000-ms-long segment time-locked to and preceding movement onset (“movement onset” class), and (2) a 1000-ms-long segment time-locked to and preceding trial start (“trial start” class). The former was expected to contain an RP-typical negativation of EEG signals at certain channels, while the latter did not contain RPs. For each segment, we subtracted the average signal in the last 200 ms of the segment from the average signal in the first 200 ms of the segment. For each segment, this value thus represented how much the signal had changed in the 1000 ms preceding either movement onset, or trial start, respectively. For each EEG-channel individually, we then performed two one-sided *t* tests to test (1) if the signal changes throughout the segment in the movement onset class were smaller than zero (to reflect the negative-going RP), and (2) if the signal in the movement onset class was smaller than that in the trial start class (to account for potential negative-going signal drifts before trial start cues). The criterion for selecting a channel was then that the null hypothesis of both these tests on the preparatory data could be rejected at an α level of 0.05. The number of selected channels thus varied between participants. Channel Cz was selected for all 22 participants, reflecting the fact that RPs preceding foot movements are typically most distinct over that channel (Brunia et al., 1985; Schultze-Kraft et al., 2016). Channels further away from Cz were selected with less frequency. On average, 10 (SEM = 1) channels were selected per participant.

#### Training of EEG classifier

In order to extract RP-related spatiotemporal features from the EEG, we performed the following analysis, using data from the preparatory stage: for each trial and each selected channel, we defined two EEG segments: (1) a 1000-ms-long segment time-locked to and preceding movement onset (movement onset class), and (2) a 1000-ms-long segment time-locked to and preceding trial start (trial start class). These segments were first baseline corrected in the interval −1000 to −900 ms and then downsampled by averaging the data in consecutive 100-ms intervals, thus obtaining 10 temporal features per segment and channel. Finally, these features were concatenated across all selected channels to obtain a spatiotemporal feature vector per segment. In order to derive an estimate of the distribution of classifier outputs for EEG segments containing RPs, we performed the following analysis: a regularized linear discriminant analysis (LDA) classifier with automatic shrinkage (Blankertz et al., 2011) was trained on the movement onset and trial start segments of all but one trial in the preparatory data, and then applied to the movement onset segment of the left out trial. This procedure was applied to each trial in a leave-one-out scheme, resulting in one classifier output value per single-trial RP. The mean μ_0_ and SD σ_0_ of the resulting distribution were calculated. These values were used during the feedback stage for transforming the EEG classifier outputs into a feedback score. Finally, the same classifier (hereafter referred to as “EEG classifier”) was trained on all trials and subsequently used during the feedback stage (see section “Real-time feedback”).

### Real-time feedback

During the feedback stage, every 20 ms both previously trained classifiers were applied to the real-time data acquired at that moment. That is, the accelerometer data acquired in the last 200 ms was subjected to the accelerometer classifier, and the EEG data acquired in the last 1000 ms was subjected to the EEG classifier. This yielded one output value per classifier at each sample point. The logic was as follows. First, we wait until the button is pressed. Then we use the accelerometer classifier to look back in time from the button press and identify the time of movement onset, defined as the classifier switching from idle to movement class. Then the EEG classifier output value at the time of this movement onset was identified. Finally, to be easily interpreted by the participants, this value *x* was transformed to a score as following:

$$score=\left(\left(x-\mu_{0}\right)/\sigma_{0}\right)\cdot 15+50.$$

That is, after being normalized by the parameters obtained from the classifier outputs in the preparatory stage, the output was transformed such that an average value would result in a score of 50, and a value being 1 SD above or below the mean would result in a score of 65 or 35, respectively. The resulting value was rounded to an integer and then showed to the participant as the feedback score after the pedal press.

### Questionnaire

After finishing the feedback stage, participants were asked to fill out a questionnaire, which consisted of four questions: (1) “Overall, how much did you feel you could influence the scores shown on screen? (1 – not at all, 5 – a lot)”; (2) “How hard/easy was it to find a strategy that had an effect on the scores? (1 – very hard, 5 – very easy)”; (3) “Please use the table on the back of this sheet to write down your experience on the strategy/strategies that you used to achieve lower scores. On the left, please describe the strategy you used. On the right, please rate the success of the strategy and comment on anything that you find worth mentioning”; and (4) “Did you have the feeling that one or more of the strategies work better over time, as if they were trainable? If so, which ones? (Please specify in the table)”.

### Data selection

Before analysis, we performed a data selection approach based on two criteria.

#### Accuracy of real-time movement onsets

We measured RPs in real time by time-locking the EEG to the time of movement onset, not the time of the pedal press. As outlined in section “Detection of movement onsets from accelerometer”, our definition of movement onset uses the accelerometer classifier and looks backwards from the button press and identifies the latest time point before pedal press that is classified as idle. However, participants might not always perform smooth and continuous movements but instead perform multiphasic movements where they briefly pause or move slowly in between. In those cases, the accelerometer classifier at times failed to detect the true time of movement onset. Therefore, we had to ensure that movements onsets were not simply a later stage of a multiphasic movement with the participant having initiated the movement much earlier. Thus, we additionally required that there was no sign of movement in the phase before the detected movement onset. We excluded trials based on the following criterion: from trials in the preparatory stage, we defined the baseline variance of the accelerometer signals during rest. A feedback stage trial was then excluded if the accelerometer signal variance in the interval from −1000 to 0 ms before the real-time assigned movement onset was three SD above the baseline, thereby excluding on average 62 (SEM = 15) trials per participant.

#### Premature movement executions

We also focused on those trials where participants adhered to the instruction to wait for roughly 2 s after trial onset before deciding to press the pedal. This was to ensure that the time window used to extract RP features from the EEG (a 1000-ms window time-locked to and preceding movement onset) did not fall into the pretrial start period. If participants did not follow this instruction, the extracted EEG features in that trial would be contaminated by the presentation of the trial start cue. Thus, we excluded trials where the delay between trial start and movement onset was <1000 ms, excluding on average 7 (SEM = 5) trials per participant.

The total number of trials excluded by these two criteria varied considerably across participants. Three participants with >50% excluded trials were excluded from all further analysis. The final sample thus included 19 participants, with an average of 255 (SEM = 9) trials.

### Statistical analysis

Four explanatory variables were defined to examine the ability of participants to alter their RPs. One variable was trial number (TN), which was the key focus in this study: if participants were successful in gradually finding and training a strategy to lower their RP feedback scores during the feedback stage, this would be reflected in a decrease of RP feedback scores as a function of TN. In addition, three additional measurements that characterize how participants generated the movement in each trial were defined as explanatory variables, despite not being the key focus here: WT (time from trial start to movement onset), movement duration (MD; time from movement onset to pedal press), and peak acceleration (PA; maximum acceleration measured between movement onset and pedal press). All four variables were z-transformed for each participant individually.

To test for an effect of the four variables on the RP, for each participant individually a linear regression was fitted on the trial-wise feedback scores (i.e., the linearly transformed EEG classifier outputs), using TN, WT, MD, PA, and a constant regressor as predictors. This yielded one estimated regression coefficient for each participant and each variable, on which we then performed one-sample *t* tests as a second-level analysis. Our main variable of interest was TN: a gradual decrease of feedback scores in the course of the feedback stage would be reflected in a negative coefficient for the variable TN. Thus, a one-sided *t* test was used to test whether the estimates were smaller than zero. For the movement characteristic variables WT, MD, and PA, we had no specific assumption about the direction of the effect. Thus, for each of these variables, a two-sided *t* test was performed. Finally, given the absence of an effect for all four variables (see Results), we validated the evidence for this absence using Bayesian *t* tests, implemented in the open-source project JASP (Love et al., 2019). The prior used for the *t* tests is described by a Cauchy distribution centered around zero and with a scale parameter of $r=\sqrt{2}/2\approx 0.707$, as suggested in Morey and Rouder (2011). Bayesian hypothesis testing aims to quantify the relative plausibility of the null and alternative hypotheses, and the Bayes factor (BF) obtained by a Bayesian *t* test is a continuous measure of evidence for either hypothesis (Keysers et al., 2020).

### Code accessibility

Analyses for this study were implemented in MATLAB, version R2016b (The MathWorks Inc.), and run on a computer using Intel core-i7 CPUs running the Ubuntu Linux operating system version 16.04. Analysis code is available by request from the authors.

## Results

### Validity of feedback scores

**Figure 2.**
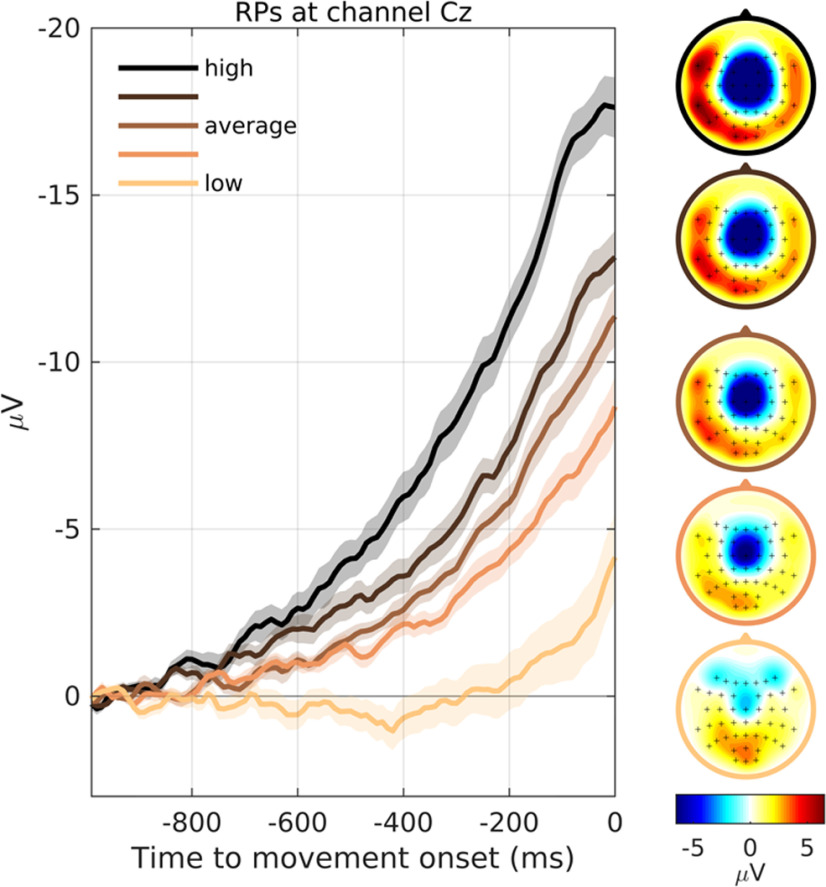
Waveforms and topographies of RPs for different feedback levels. For each participant, trials were grouped into five quantiles depending on the feedback score that was calculated in real time, color coded in all panels from low (light) to high (dark). The left shows the grand average waveforms of RPs at channel Cz for the five quintiles, baseline corrected in the interval [−1000,−900] ms. SE is shown as a shaded area. The right shows the corresponding scalp topographies of the average voltage in the interval [−100,0] ms for the five quintiles.

We first verified that the feedback scores presented to participants indeed reflected the size of the RP (as would be expected by our method for defining feedback). Figure 2 shows average RPs for the five different quintiles of feedback scores (low to high). RPs with high scores had early onsets and high amplitudes, whereas RPs with low scores had late onsets and small amplitudes. While RPs at all score levels had their largest amplitudes at the vertex (channel Cz), they were spatially less pronounced in more distant electrodes at lower scores. The correlation between feedback score and RP amplitude was confirmed with a mixed-effects regression (*β* = −1.434, *p *<* *0.001). Thus, on average, a decrease in 1.4 units of feedback score was equivalent to a 1-μV decrease in RP amplitude.

### Manipulation of scores by participants

**Figure 3.**
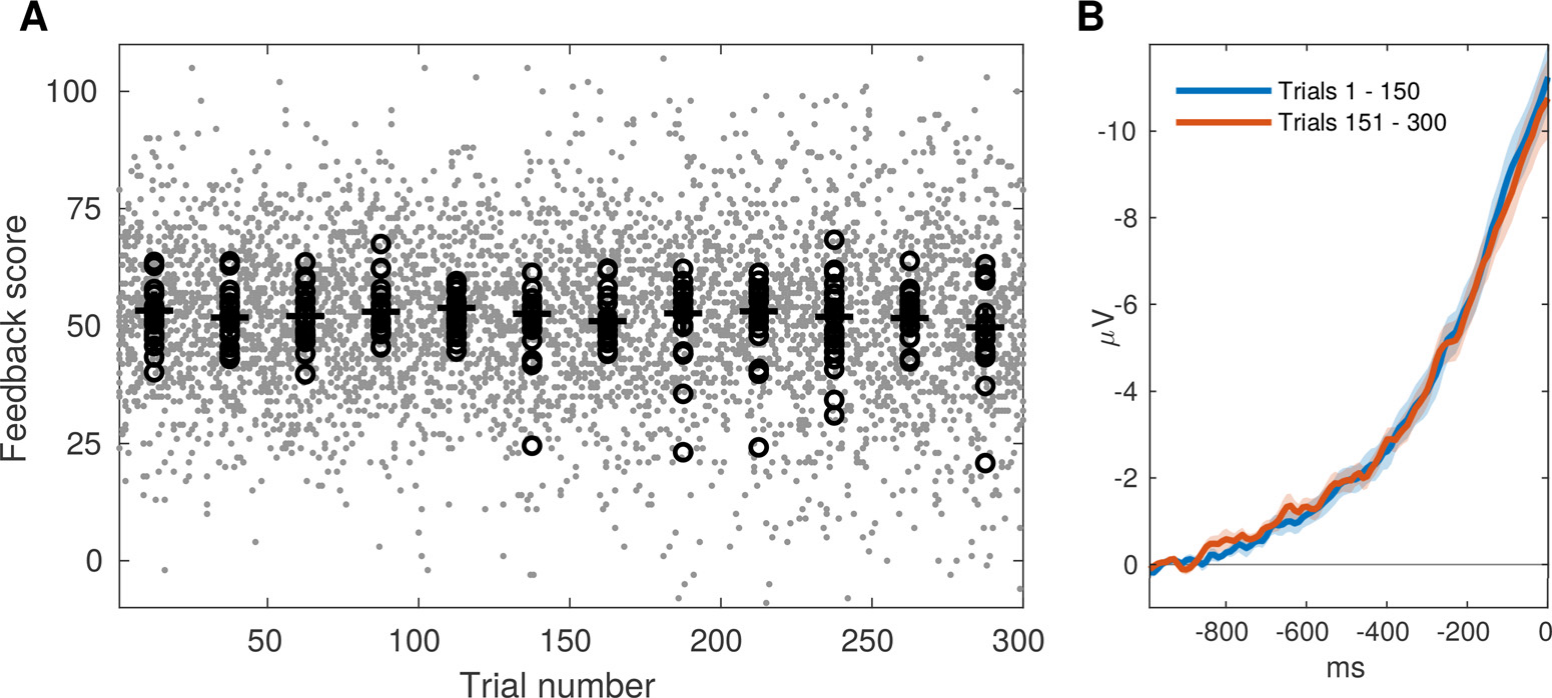
Change of feedback scores and RP waveform in the course of the feedback stage. ***A***, Gray dots show single-trial feedback scores, pooled across all participants, as a function of TN. Black circles and vertical lines show averages of individual participants and population medians, respectively, both calculated over consecutive, non-overlapping bins of 25 trials. ***B***, Grand average RPs in channel Cz, computed for the first (blue) and the second (red) half of trials, respectively. SE is shown as a shaded area. Baseline correction was in the interval −1000 to −900.

We examined whether participants were successful in finding a strategy to execute movements with lower scores. If they were, this should be reflected in a gradual decrease of scores over the course of the 300 trials. A visual inspection of scores as a function of TN showed no indication of such decrease, and the shape RP waveform did not change over time (Fig. 3). A one-sided *t* test on the regression coefficient estimates obtained for each participant showed that they were not smaller than zero (*t*_(18)_ = 0.103, *p* = 0.541), and the BF_0-_ = 4.539 indicates that the data are 4.5 times more likely under the null hypothesis which provides moderate evidence for absence (Jeffreys, 1961) of an effect of TN. The lack of a negative (linear) trend of feedback scores during the 300 trials of the feedback stage suggests that participants were not successful in finding a strategy to willfully reduce their RPs.

**Figure 4.**
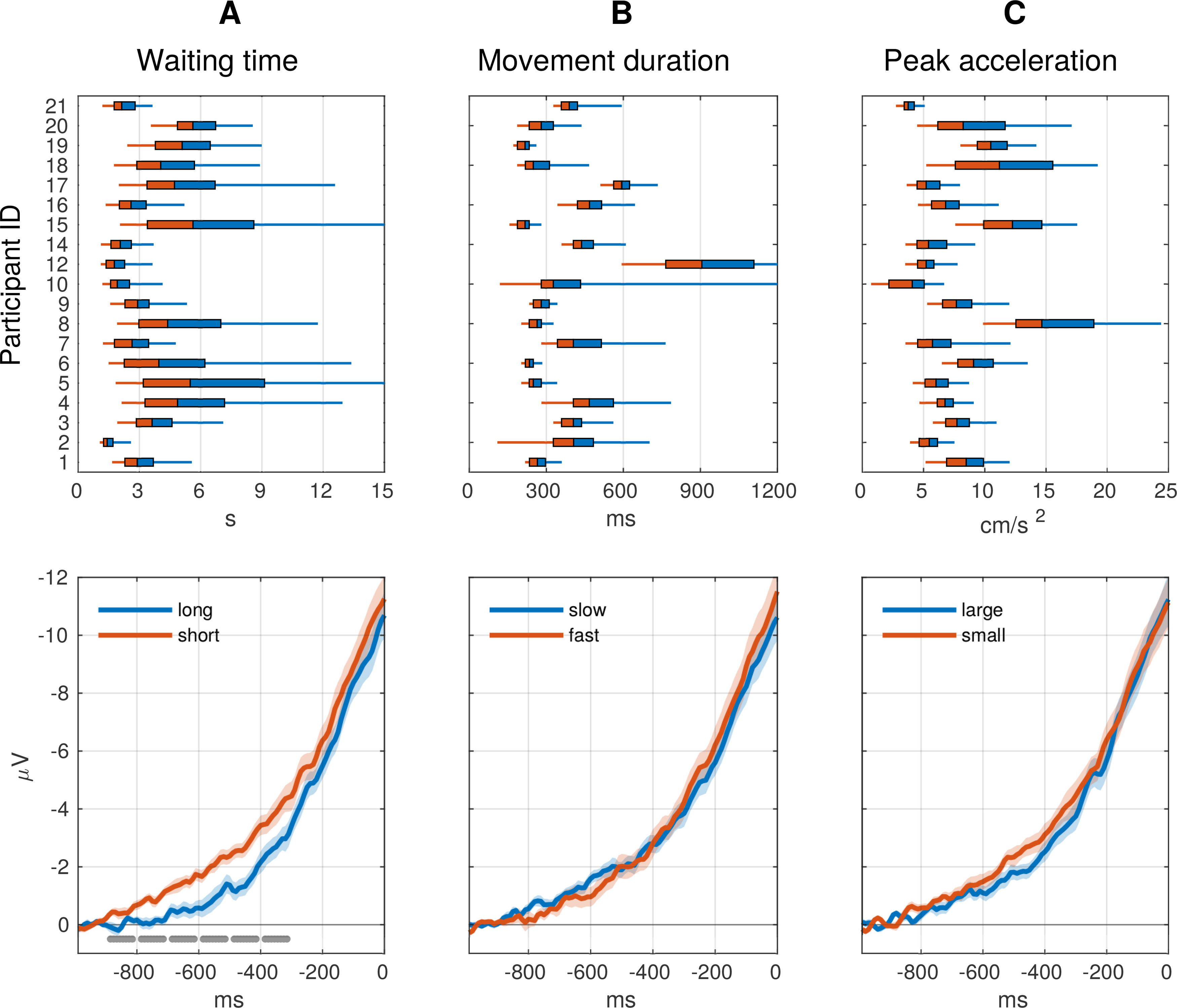
Modulation of RPs by movement characteristics (channel Cz). We checked whether basic spontaneous movement characteristics included in the model as effects of no interest modulated the RP waveforms (as reported in previous literature). Top, Boxplots show, for each participant individually, the distribution of WT (***A***), MD (***B***), and PA (***C***) of movements executed. Bottom, For each participant individually, two average RPs were generated each using half of the trials (according to a median split of the corresponding measure, indicated in the color coded regions of the boxplots). The curves show grand averages across participants. Blue and red traces show the average RP for the shorter and longer half of WT (***A***), for the faster and slower half of movements (***B***), and for the smaller and larger half of PAs (***C***), respectively. SE is shown as a shaded area. Baseline correction was in the interval −1000 to −900. Gray bars indicate in which consecutive, non-overlapping 100-ms windows a paired *t* test showed a significant (*p* < 0.05) difference of RP averages. The small differences in the waveform in ***A*** did not affect the EEG classifier (and therefore the feedback scores), which takes into account the RP waveform across all selected channels.

Next, we examined whether the RP was modulated by either of the three movement characteristics WT, MD, and PA. A visual comparison of RPs averaged according to a median split of the three measures of movement characteristics showed only minor differences (Fig. 4), as compared with the inherent variability of RP waveforms (Fig. 2). There is an apparent small difference in early time periods between short and long WTs (Fig. 4*A*) that is not detected as significant in our regression analysis. Our data do not allow us to tell whether this is a spurious effect because any testing of this time period would be *post hoc*. Two-sided *t* tests on the regression coefficient estimates obtained for each participant showed that they were not significantly different from zero (WT: *t*_(18)_ = −1.121, *p* = 0.277; MD: *t*_(18)_ = −0.373, *p* = 0.713; PA: *t*_(18)_ = 1.114, *p* = 0.279). BFs for all three variables (WT: BF_01_ = 2.432; MD: BF_01_ = 3.955; PA: BF_01_ = 2.448) furthermore show that the data are more likely under the null hypothesis and indicate a moderate evidence for absence of an effect. Thus, these results suggest the absence of a relationship between RPs and the range of movement parameter variation observed in this study. Please note that the effects of these variables are not of interest for our core research question because they are (1) not used by the participants to improve their scores and (2) reflect physical changes in the movements.

### Self-assessment of task

**Figure 5.**
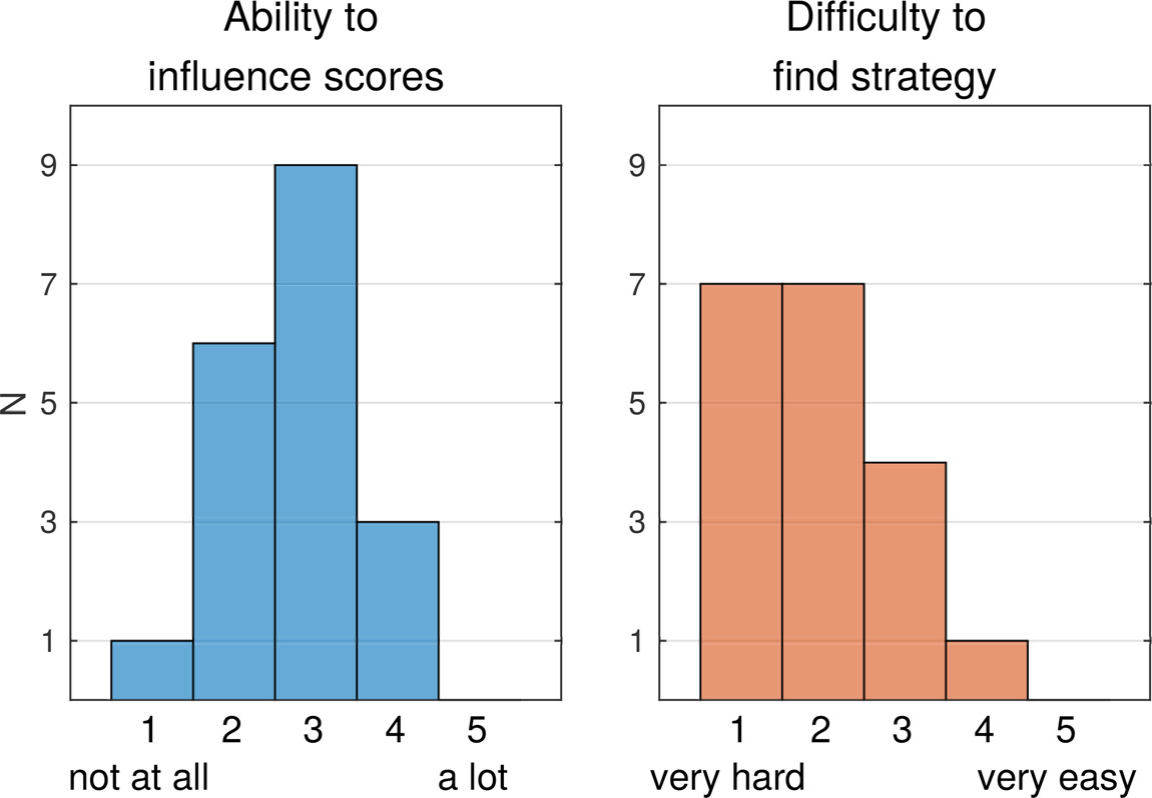
Likert scale rating in questionnaire of subjective experience. Histograms show the number of participants rating question 1 (“Overall, how much did you feel you could influence the scores shown on screen?”) on a Likert scale from 1 (not at all) to 5 (a lot), and question 2 (“How hard/easy was it to find a strategy that had an effect on the scores?”) on a Likert scale from 1 (very hard) to 5 (very easy).

We used a questionnaire after the feedback stage to assess participants’ experiences and strategies (Fig. 5). When asked to rate how much they felt they could influence scores, the most frequent rating was 3 (“average”). When asked to rate how difficult it was to find a strategy that had an effect on the scores, the most frequent ratings were 1 and 2 (“very hard” and “rather hard”). Participants were also asked to describe in written form the used strategies and whether they were successful. Among the strategies reported as “successful” or “partly successful,” participants named strategies involving attention/relaxation (11), changing the physical attributes of the movement such as speed or force (5), changing WT (4), and involving emotion (3). For detailed reports of participants’ answers, please see the Extended Data Figure 1-1.

10.1523/ENEURO.0425-20.2020.f1-1 Extended Data Figure 1-1 Participants΄ self-reports on questionnaire about strategies. Download Figure 1-1, DOCX file.

## Discussion

Can people learn to suppress their RP? We tested this possibility in a neurofeedback experiment. Participants performed self-paced pedal presses in single trials, and after each pedal press, they were provided with a feedback score that reflected the magnitude of the RP preceding that movement.

To extract the scores from RPs, we employed a machine learning approach: we used data acquired during a preparatory stage to train a classifier to distinguish EEG segments preceding movements from the idle period before onset of the trial. By extracting spatiotemporal features from the EEG, the classifier learned both the spatial distribution of the RP across channels, and the characteristics of its waveform. Thus, when the classifier was applied to an EEG segment preceding a movement onset during the feedback stage, the resulting classifier output, and thereby the feedback score, was a continuous indicator of the degree to which an RP was present in that segment (Fig. 2).

Participants were challenged to find a way to perform self-paced movements with small RPs, and were instructed to keep using and extending any potentially effective strategies. If they were successful, this would be reflected in a gradual decrease of feedback scores in the course of the 300 trials of the feedback stage. However, we found no evidence for such a decrease (Fig. 3), suggesting that participants were not able to find or train a successful strategy.

This finding does not rule out the possibility that participants were able to occasionally modulate their RPs. Possibly some weaker potential effects of their strategies went unnoticed and were thus not further explored. One way to test this is to examine the relationship between feedback scores and the movement characteristics that participants were able to modulate and that we could measure in every trial: how long participants waited from trial start until initiating the movement (WT), how fast they executed the movement (MD), and how much force they applied to the movement, as reflected by the PA during movement execution. Although WT slightly modulated the shape of the RP (Fig. 4*A*), we found no evidence for an effect of either of the three movement parameters on the feedback score.

The failure to deliberately suppress the RP does not reflect a fundamental impossibility that small RPs occur. Our data clearly show that many RPs recorded during the feedback stage were remarkably small in size: one out of five movements were preceded by RPs with very late onsets of only a few 100 ms, had amplitudes >50% smaller as compared with the average, and a substantially more confined spatial distribution (Fig. 2, light color code). These small RPs occurred with a fairly constant rate throughout the feedback stage (Fig. 3). We cannot fully exclude the possibility that the small amplitude of these RPs was somewhat influenced by mental strategies; however, if so, then participants failed to notice or systematically exploit the effects of these strategies.

When asked how difficult it was to find a successful strategy, most participants (14 out of 19) reported it was hard or very hard (Fig. 5). This self-assessment is in agreement with our finding that scores did not decrease over the course of the feedback stage. Interestingly, there appears to be some kind of “illusion of control”: when asked to rate their general ability to influence scores, participants’ ratings peaked at the midpoint of the scale, reflecting a “moderate” perceived ability to influence scores. In the absence of true control over RP scores this could suggest alternative interpretations. Possibly participants were biased to remember more of those trials in which an intended strategy happened to coincide with a purely random low score, and less those in which the effect was contrary (Thompson et al., 1998). Alternatively, people often tend to choose scores in the middle of Likert scales (which is known as the central tendency bias), particularly when they are unsure about their answer (Nadler et al., 2015). Thus, the predominance of central ratings in this question could be interpreted as participants meaning “I don’t know,” rather than as reporting a perceived ability to influence scores.

Finally, it is worth noting that we cannot exclude the possibility that participants were effectively able to slightly modulate their RPs, for instance by employing strategies based on attention which we did not measure and therefore could not test. It has been shown that RPs are smaller when spontaneous movements are initiated unconsciously, that is without attention (Keller and Heckhausen, 1990; Takashima et al., 2018; Houdayer et al., 2020). Furthermore, Birbaumer (1999) suggested that learning the self-regulation of SCPs is based on a “redistribution of attentional resources.” Interestingly, when we asked our participants to describe strategies that (partly) worked, the most frequently reported strategy involved attention (Extended Data Fig. 1-1). However, even if participants were effectively able to slightly modulate their RPs by employing strategies based on attention, this modulation effect was too small for participants to train it or to sustain it over longer periods. This is possibly because of the inherent contradictoriness of the intent to attentively (and constantly) shift attention away from a task.

At first sight, our data seem to differ from previous findings that paralyzed patients can learn to self-regulate their SCPs by means of real-time visual feedback (Kübler et al., 1999, 2001; Neumann et al., 2004). This raises the question why participants in our study were not able to use comparable mechanisms to suppress their RPs. We consider two potential reasons: first and foremost, in those studies paralyzed patients learn and train the task of SCP self-regulation in multiple sessions over the course of several weeks or months. While such an approach would be prohibitively expensive for the purposes of this study, it is conceivable that learning our task might be possible if participants were to be provided with much more time. Thus, future research is required to assess whether people can learn to exert control over their RPs using longer training protocols. Second, SCPs investigated in those studies reflect changes in cortical polarization that occur spontaneously in the ongoing EEG. In contrast, RPs are defined as time-locked to the onset of a voluntary movement, and it has been recently debated whether they occur in the absence of voluntary action (Travers et al., 2020). Thus, it is possible that the event-related nature of RPs impedes their conscious self-regulation by the mechanisms through which other SCPs are influenced. Finally, it is worth noting that we deliberately did not provide participants with any specific instructions as to how they could achieve the task of suppressing their RPs. We abstained from doing so because we did not make specific assumptions about whether or how this task was possible. Thus, we aimed to test whether a trial-and-error approach was sufficient for participants to find a successful strategy, without introducing a bias on potential strategies. It is however possible that providing specific instructions for mental strategies might have facilitated participants to identify and train a successful strategy.

Our data confirm and expand findings from a recent study, where stop signals were elicited in real time on detection of RPs while participants were performing self-initiated movements (Schultze-Kraft et al., 2016). In one condition, participants were instructed to “move unpredictably” so as to not cause stop signals. However, the shape of the RP remained unchanged and stop signals thus continued to be elicited, suggesting that participants were unsuccessful in reducing or suppressing their RPs. In that study, to avoid stop signals being triggered by noise in the EEG, they were only elicited if the magnitude of an RP was above a certain threshold. Thus, those stop signals can be considered a binary feedback of the RP, since they were triggered by large but not by small RPs. In contrast, in the current study, the feedback of the RP was continuous: in every trial, a feedback score was shown that directly reflected RP magnitude on a continuous scale. The trial-by-trial feedback in this study thus provided considerably more information about the RP to the participant, compared with the binary stop signals used in our previous study (Schultze-Kraft et al., 2016). However, the data of both studies suggest that the inability of participants to exert control over their RPs does not depend on the type or scale of the provided feedback.

Our main finding that participants were not able to consciously suppress or modulate their RPs suggests that the RP is a signal over which people cannot exert conscious control, and thus that it is an “involuntary precursor signal of voluntary action.” Please note, however, that our data remain silent as to whether the RP is a causal precursor signal of voluntary action, as has been the traditional account of the RP (Libet et al., 1983; Libet, 1985). Alternative accounts suggest that the RP reflects the leaky integration of spontaneous fluctuations in a drift-diffusion process (Schurger et al., 2012; Schurger, 2018), and that spontaneous movements occur when the accumulation of autocorrelated noise reaches a threshold, with either the output (Schurger et al., 2012) or the input (Schurger, 2018) of this accumulation giving rise to the shape of the RP.

The accumulation-to-bound model makes several predictions relevant for the interpretation of our data. First, the RP-as-input model (Schurger, 2018) predicts that the shape of the RP is influenced by the delay between trial start and movement onset, i.e., the WT. Indeed, visual inspection of our data show a slight modulation of RP waveform in channel Cz by WT (Fig. 4*A*), compatible with the report by Schurger (2018; their Fig. 6). Note, however, that this modulation is not detected as significant in our regression analysis on the feedback scores, where the BF supports the absence of an effect. This is possibly because our EEG classifier was trained in a more robust fashion on changes in RP across all channels selected in the training data. Thus, it is unclear whether this effect is spurious. Second, in the accumulator model framework people could potentially exert influence over their RPs, e.g., by modulating parameters such as drift rate or threshold, as long as these were in turn to change the shape of the RP. Nonetheless, if this accumulation is necessary for voluntary movements then such movements would necessarily be preceded by an RP. Our data show that participants seem unable to affect the amplitude of the RP, even when explicitly trying to do so.

In sum, we performed a neurofeedback experiment to test whether people are able to suppress their RP. We found no evidence for the ability of participants to consciously suppress their RPs. Our findings thus suggest that the RP is an involuntary precursor signal of voluntary action over which people cannot exert conscious control.

## Synthesis

Reviewing Editor: Alexxai Kravitz, Washington University in St. Louis Decisions are customarily a result of the Reviewing Editor and the peer reviewers coming together and discussing their recommendations until a consensus is reached. When revisions are invited, a fact-based synthesis statement explaining their decision and outlining what is needed to prepare a revision will be listed below. The following reviewer(s) agreed to reveal their identity: Karim Fifel.

We have four main comments:

1) To comply with eNeuro policy, you should include in the Materials and Methods section under the heading "Code Accessibility" whether and how the code can be accessed, including any accession numbers or restrictions, as well as the type of computer and operating system used to run the code.

2) Both reviewers thought the figures could be condensed and some could be removed to streamline the presentation of the results. We thought it would be possible to combine them into a total of 4-5 figures, which would make it easier for a reader to grasp the main points. Some suggestions for this:

a. Figures 1 and 2 could be combined

b. Figure 3 and 4 are not strictly necessary and could be removed, with their main points explained in the text

c. Figure 6 could be summarized and appended as a panel to another figure

d. Figures 7 and 8 could be combined

e. Figure 9 could be removed and summarized in text, or a summary analysis could be appended into a panel in the previous figure

f. Figures 10 and 11 could be combined

3) The authors should better discuss the rationale behind the study. Specifically, the authors should discuss the specific training protocol they used, and how their results might have differed if longer training protocols were used. For example, if a long-term, rehabilitation-style, experiment were completed, would the authors expect patients to be able to modulate the RP? Do their results speak to this?

4) The authors may want to consider adding a visual abstract.

## Author Response

Reply to reviewer #1

Readiness potential (RP) is a potential that precedes a deliberate act by approximately 600ms. Since the seminal work of libet that pointed out that most of the RP emerges before the subject is aware of the urge to move, controversies as to the meaning of the RP persisted.

The current study did not aim to investigate any behavioural or physiological correlate of the RP. However, the authors tried to investigate whether it is possible to influence consciously the amplitude of the RP.

Methodologically, the study is well conducted with nice control experiments. The authors used a feedback visual input depicting an estimate of the amplitude of the RP to instruct the participants to find a strategy to modulate the RP.

The study showed that it was not possible for the participant to modulate the characteristics of the RP, hence the nicely chosen title of the article.

We thank the reviewer for these kind comments.

The main issue that needs further discussion in the paper is related to the rational behind the study. The authors based their assumption on the well-established studies showing the possibility of modulating slow cortical potentials (SCP). However, a critical point in relation to SCP studies is the relative contribution of conscious vs subconscious factors as causal modulators of the SCP.

Let us consider the following;

1) We know that RP emerge subconsciously.

2) We know that subconscious factors (i.e. visual stimuli) can influence both brain activity and related behaviour (Shibata et al., 2011, Science).

3) What option(s) do participants have to modulate the amplitude of the RP? As the authors said, presumably ‘Distribution of attentional resources’.

Given 1, 2, 3 and the short duration of the experiment, would we expect a different result that the one obtained? Otherwise, would a long-term, rehabilitation-style, experiment lead to different outcome? Namely, Yes we can modulate the RP.

We thank the reviewer for bringing up this important point.

Indeed, the traditional view of the RP (most notably put forth by Libet in his seminal work, 1983, 1985) has been that it is a preconscious precursor to voluntary movement. If the RP is initiated preconsciously, it would indeed be questionable if and how people would be able to exert control over their RPs. On the other hand, this view has been recently challenged (e.g. Schurger et al., 2012), suggesting that the "preconscious" component of the RP is but an averaging artifact, and that the RP component related to movement preparation occurs later, close to the time of the conscious decision to move. Thus, despite possible interactions betwen unconscious factors and behavior, it remains conceivable for people to be able to modulate their RP.

Regarding the possible mechanisms that participants have, early studies on SCP modulation have suggested a "redistribution of attentional resources", although this is not well understood. Early work by Keller and Heckhausen (1990), but also more recent similar studies (Takashima et al., 2018; Houdayer, Lee & Hallett, 2020), have indeed shown that unconsciously initiated spontaneous movements (i.e. without attention) are preceded by smaller RPs. As a matter of fact, in the perticipants’ self-reports of strategies that "worked well" or "worked sometimes" (Extended Data Table 1-1), the most frequently reported strategy involved attention. Nonetheless, our data show that this strategy was not sufficient for participants to successfully, robustly and persistently reduce their RPs. This is possibly because the task itself required a permanent and sustained high level of attention. We now realize that this point was not sufficiently clear and have now modified/added the following to the discussion:

"Finally, it is worth noting that we cannot exclude the possibility that participants were effectively able to slightly modulate their RPs, for instance by employing strategies based on attention which we did not measure and therefore could not test. It has been shown that RPs are smaller when spontaneous movements that are initiated unconsciously, that is without attention (Keller & Heckhausen, 1990; Takashima et al., 2018; Houdayer, Lee & Hallett, 2020). Furthermore, Birbaumer (1999) suggested that learning the self-regulation of SCPs is based on a "redistribution of attentional resources". Interestingly, when we asked our participants to describe strategies that (partly) worked, the most frequently reported strategy involved attention (Extended Data Table 1-1). However, even if participants were effectively able to slightly modulate their RPs by employing strategies based on attention, this modulation effect was too small for participants to train it or to sustain it over longer periods. This is possibly due to the inherent contradictoriness of the intent to attentively (and constantly) shift attention away from a task." (Page 17)

As rightly pointed our by the reviewer, it is possible that using a longer training protocol in our study could have potentially enabled participants to learn how to suppress their RPs. In the early work on self-regulation of SCPs, participants learned that taks in long-term, rehabilitation-style experiments over several months. Future research is required in order to assess whether it is indeed not possible for people to exert control over their RPs, or whether they can learn this using longer training protocols. We have modified the text in the discussion to make this more clear:

"First and foremost, in those studies paralyzed patients learn and train the task of SCP self-regulation in multiple sessions over the course of several weeks or months. While such an approach would be prohibitively expensive for the purposes of this study, it is conceivable that learning our task might be possible if participants were to be provided with much more time. Thus, future research is required in order to assess whether people can learn to exert control over their RPs using longer training protocols." (Page 17)
