# Supplementary material for: Suppress Me if You Can: Neurofeedback of the Readiness Potential
Source: eNeuro. 2021 Mar 8;8(2):ENEURO.0425-20.2020. doi: 10.1523/ENEURO.0425-20.2020 (PMC7986527; doi:10.1523/ENEURO.0425-20.2020)
Supplement: Extended Data Figure 1-1 — Participants΄ self-reports on questionnaire about strategies. Download Figure 1-1, DOCX file. [file enu-eN-NWR-0425-20-s02.docx]

**Extended Data**

**Participants’ self-reports on questionnaire about strategies**

**Figure 1-1. Participants’ self-reports on questionnaire about strategies.** Participants were asked to write down (i) the different strategies that they used during the feedback stage, (ii) whether they worked, and if so (iii) whether they were trainable. The table shows all reported strategies of single participants in condensed form. Participants’ written accounts of how well each strategy worked were classified into three rating categories (right column): accounts such as “unsuccessful” or “did not work” are denoted with a 0, accounts such as “seemed to work” or “worked sometimes” are denoted with a +, accounts such as “successful” or “worked well”, or those reported as trainable, are denoted with a ++.

**Participant**

**ID**

**Strategy Rating**

1 Adjust breathing 0

Focus on the impulse to press **+** Shift attention to something else **+** Relax 0

2 Focus on the visual stimulus 0

3 Plan the next movement 0

Ignore the goal 0

Reward myself for good scores 0

4 Relax and clear the mind **+** Evoke positive emotions **++** Ignore scores 0

5 Relax, don’t think about pressing **++**

Move faster 0

Think about low numbers 0

6 Clear the mind **+**

Play song in my head and hit on specific beats 0

Delay the first urge to press and press later 0

7 Don’t think about pressing **+** Focus on pressing **++** Delay first urge to press and press later **+**

Feel surprised about pressing the pedal **++**

8 Slow down movement and decrease force **+**

9 Delay the urge to move and move shortly after **++** Don’t think about pressing **+** Longer waiting time 0

10 Focus on stimulus or task 0

Focus on breathing, clearing the mind 0

Slower and less forceful movements **++**

Longer waiting time 0

11 Longer/shorter waiting time 0

Increase/decrease force of movement 0

Interrupting movement **++**

12 Relax 0

Slower movement **++**

13 Slower movement **++**

Decrease force of movement 0

Decrease waiting time 0

14 None specified 0

15 Mind wandering (use spontaneous train of thoughts) **++** Relax **+** Shifting attention away from task **+** Using emotions **+**

16 Increase force of movement **++** Shift attention away from task **+** Increase waiting time 0

Relax **+**

17 Clear mind, relax **+**

Move spontaneously **+**

18 Think about something else 0

Focus on breathing **+** Focus on replicating the same movement every trial **++** Shift attention away from task 0

Increase movement speed 0

19 Shift attention away from task **+**

20 Waiting time **++**

Force of movement 0

Movement speed **++**

21 Moaning **+**

Relax 0

Speed of movement 0

22 Delay the first urge to move, move later **+**
